# Mitochondrial Genome and RNA Editing Tissue Specificity of *Centella asiatica*

**DOI:** 10.3390/genes16080953

**Published:** 2025-08-12

**Authors:** Cuihong Yang, Wenjing Liang, Ya Qin, Yuqiong Li, Shugen Wei, Qiulan Huang, Ahmed H. El-Sappah, Guiyu Tan, Ying Wei, Lingjian Gui, Lingyun Wan

**Affiliations:** 1 Guangxi Key Laboratory for High-Quality Formation and Utilization of Dao-di Herbs, Guangxi Botanical Garden of Medicinal Plants, Nanning 530023, China; 2 National Center for Traditional Chinese Medicine (TCM) Inheritance and Innovation, Guangxi Botanical Garden of Medicinal Plants, Nanning 530023, China; 3 National Engineering Research Center for the Development of Southwestern Endangered Medicinal Materials, Guangxi Botanical Garden of Medicinal Plants, Nanning 530023, China; 4 Faculty of Agriculture, Forestry and Food Engineering, Yibin University, Yibin 644000, China

**Keywords:** *Centella asiatica*, mitochondrion, RNA editing

## Abstract

Background: *Centella asiatica*, a medicinally important species that is rich in bioactive compounds, lacks a characterized mitochondrial genome, despite nuclear and chloroplast assemblies. We sequenced and annotated its mitochondrial genome to elucidate its genetic foundations and evolutionary mechanisms. Methods: Assembly using Illumina short-reads and Nanopore long-reads was used to characterize the mitochondrial genome. Analyses included structural characterization, codon usage bias, repetitive sequences, horizontal gene transfer (HGT), collinearity, and phylogeny. The resulting tissue-specific (root, stem, and leaf) long non-coding RNA (lncRNA) profiles identified RNA editing sites. Results: The complete mitochondrial genome (249,777 bp, 45.5% GC) comprises three circular contigs encoding 51 genes (33 protein-coding, 15 tRNA, and 3 rRNA). Comparative genomics revealed synteny with the Apiaceae family of plants and evidence of HGT. Phylogenetic analysis resolved taxonomic relationships within Apiales. We predicted that 547 RNA editing sites would be identified in its protein-coding genes. Tissue profiling identified 725 (root), 711 (stem), and 668 (leaf) editing sites, with >71% concordance to predictions. RNA editing-generated cryptic promoters/terminators occur in mitochondrial core function genes (e.g., ATP synthase, cytochrome c reductase/oxidase, ribosome large subunit, and cytochrome c biogenesis), exhibiting a lower frequency in the leaves compared to the roots and stems. Conclusions: We provide the first complete mitochondrial genome assembly for *C. asiatica*, delineating its complex structure, tissue-modulated RNA editing, and evolutionary trajectory. This high-quality genomic resource establishes a foundation for molecular evolutionary studies and enhances the genomic toolkit for this pharmacologically significant species.

## 1. Introduction

*C. asiatica*, a perennial herbaceous species in the Apiaceae family, exhibits a pantropical distribution pattern and predominantly occurs in shaded mesic microhabitats at elevations below 2000 m [1]. Renowned for its pharmacological potency, this species accumulates diverse bioactive triterpenoids, particularly asiaticoside and madecassoside, which underpin its therapeutic applications in managing jaundice, heatstroke, fever, diarrhea, urinary calculi, hematuria, carbuncles, and wounds [2]. Such validated ethnopharmacological utility has secured its official recognition in multiple pharmacopeial monographs across Asian and European regulatory frameworks [3,4]. Despite considerable advances in *C. asiatica* genomics, current knowledge is mainly focused on its nuclear and chloroplast genomes, with the mitochondrial genome being persistently uncharacterized. This critical gap impedes our systematic understanding of mitochondrial and nuclear genomic coordination in triterpenoid biosynthesis and obscures evolutionary trajectories within the Apiaceae family. Fundamental questions remain unresolved: What genomic structures and features does the *C. asiatica* mitochondrial genome possess? How do its repetitive sequence components differ from those of other plants? What environmental adaptation mechanisms do these differences reflect? What implications might these findings have for the molecular breeding of medicinal plants? These knowledge gaps collectively underscore the necessity for de novo assembly and functional annotation of the *C. asiatica* mitochondrial genome. A high-resolution mitochondrial genomic resource would not only clarify the species’ organellar genome evolution but also enable multi-omics integration to decipher how mitochondrial–nuclear crosstalk modulates stress-responsive secondary metabolism.Plant mitochondria are essential organelles that are primarily responsible for cellular respiration and energy production. The mitochondrial genomes display extraordinary size variation in angiosperms, spanning three orders of magnitude across the taxa, from compact genomic lengths of 101–141 kb in *Buxbaumia* species to the colossal 11.7 Mbp mitochondrial DNA length documented in *Larix sibirica* Ledeb [5,6]. These genomic fluctuations originate from synergistic interactions among multiple evolutionary drivers, including the accretion of non-coding DNA, the proliferation of repetitive sequences, and the integration of exogenous DNA, along with the gain or loss of large genomic fragments within mitochondrial genomes [7]. Contrary to the canonical circular paradigm, plant mitochondrial genomes demonstrate considerable complexity, adopting a dynamic array of topological conformations that include linear assemblies and multibranched chromosomal structures, which may exist as single or multiple segments. For instance, *Haloxylon ammodendron* possesses two complete circular mitochondrial DNA structures, whereas *Ventilago leiocarpa* exhibits a quadripartite multibranched genome wherein coting1 (ctg1) can form either an independent circle or combine with ctg4 to create linear molecules, ctg2 demonstrates similar structural plasticity, and ctg3 maintains a circular conformation [8,9]. Another study elucidated the mitochondrial genome of *Panax notoginseng*, revealing the dynamic interconversion between a “main circle” and multiple “sub-circles”, mediated by recombination events associated with DNA replication-related repeats [10]. Wang et al. emphasize that plant mitochondrial genomes are highly dynamic, featuring widespread recombination among repeats, multipartite configurations, and frequent DNA exchanges that reshape genome architecture across angiosperms [11,12]. They further argue that the mutation landscape of plant organellar DNA is shaped by recombination-driven rearrangements and pervasive RNA editing, prompting a reevaluation of longstanding mutation hypotheses for mitochondrial genomes. These phenomena collectively underscore the extraordinary dynamism inherent in plant mitochondrial genome configurations, where repeat sequence-mediated recombination mechanisms act as the principal architect of conformational changes. Notably, substantial mitochondrial genome structural variations occur, even within the genus levels. For example, *Populus tremula* contains a singular circular mitochondrial genome, whereas its congeners *Populus deltoides* and *Populus simonii* possess tripartite circular genomes [13]. Such combinatorial complexity, when compounded by frequent intramolecular recombination, poses formidable challenges for de novo mitochondrial genome assembly.

RNA editing serves as a fundamental post-transcriptional regulatory mechanism in plant mitochondrial gene expression that is predominantly characterized by cytosine-to-uracil (C-to-U) conversion events. The frequency of these editing sites exhibits remarkable evolutionary divergence across species and developmental regulation between tissues. Comparative analyses reveal extreme variations in RNA editing site abundance, ranging from the minimal 11 edited loci in *Physcomitrella patens* mitochondria to the 865 sites identified in *Nymphaea* [14,15]. Systematic investigation of *Nicotiana tabacum* through multi-tissue RNA-seq profiling (roots, stems, leaves, and flowers) delineated tissue-modulated editing dynamics, identifying 204, 294, 234, and 343 RNA editing sites in these respective tissues, with 106 strictly compartmentalized sites across 11 mitochondrial genes. Comparative analysis of three tobacco cultivars revealed editing related to the root-specific attenuation of RNA (particularly affecting the ndh genes). This suppression mechanistically correlated with both diminished average editing efficiency and the transcriptional downregulation of MORFs (multiple organellar RNA editing factors) and pentatricopeptide repeat (PPR) proteins, establishing the centrality of trans-factor availability in spatiotemporal editing regulation [16]. Complementary transcriptomic evidence from *Corydalis saxicola* mitochondria revealed the diminished expression of 24 core mitochondrial protein-coding genes (PCGs) in the roots and flowers compared to aerial tissues, including stems, branches, mature leaves, and young leaves [17]. These expression differences may indirectly reflect the widespread variation found in RNA editing sites across tissues, implying that RNA editing could serve as an evolutionary tuning mechanism to tissue-specific energetic demands.

Recent technological breakthroughs in long-read sequencing and strand-specific RNA sequencing have provided crucial technical support for advancing plant mitochondrial research. The advent of long-read sequencing platforms enables the precise resolution of complex tandem repeats and low-complexity regions that have traditionally confounded accurate mitochondrial genome assembly [18]. Meanwhile, strand-specific lncRNA sequencing provides dual analytical capacity, not only for the high-confidence detection of C-to-U RNA editing sites through directional read mapping but also for revealing cis-regulatory networks mediated by antisense transcripts in mitochondrial RNA metabolism [19]. Capitalizing on these advancements, we report the first de novo assembly and annotation of the mitochondrial genome of *C. asiatica* by integrating Nanopore and Illumina sequencing data. Comprehensive analyses were performed, including structural characterization, repetitive sequence annotation, gene content profiling, RNA editing site identification, homologous sequence analysis, collinearity assessment, and phylogenetic reconstruction. Furthermore, tissue-specific RNA editing sites in the roots, stems, and leaves were systematically identified using lncRNA sequencing data. These findings will serve as a valuable resource for future studies on the genetic evolution and mitochondrial gene expression of this species.

## 2. Materials and Methods

### 2.1. Plant Material Acquisition and Sequencing

Fresh *C. asiatica* leaves were collected from the Guangxi Medicinal Botanical Garden research base in Nanning, China (22°51′ N, 108°23′ E). Voucher specimens (Collection ID: wly2021) were preserved and kept in the research group of the current author, Dr. Lingyun Wan, at the Guangxi Botanical Garden of Medicinal Plants. Specimens were morphologically authenticated by Dr. Lingyun Wan and stored at −80 °C, with the corresponding genomic data deposited in the NCBI SRA under accessions SRR34771537 to SRR34771541. Genomic DNA extraction and quality validation followed Jianfeng et al.’s methodology [20]. Oxford Nanopore Technology (ONT) libraries were prepared using the SQK-LSK109 kit (Oxford Nanopore, Oxford, UK), with subsequent loading onto R9.4 Spot-On Flow Cells (Oxford Nanopore, Oxford, UK) and sequencing performed by Benagen (Wuhan, China) [21]. Paired-end Illumina libraries (150 bp insert size) were constructed using the Nextera DNA Flex Library Prep Kit (Illumina, San Diego, CA, USA) and then sequenced on an Illumina NovaSeq 6000 platform (Illumina, San Diego, CA, USA) [22].

### 2.2. Mitochondrial Genome Assembly and Annotation

A hybrid strategy combining Illumina (San Diego, CA, USA) and Nanopore (Oxford, UK) sequencing was used to assemble the *C. asiatica* mitochondrial genome [23]. The GetOrganelle program (v1.7.5, using default parameters) was employed to reconstruct a graphical map of the plant mitochondrial genome from second-generation DNA sequencing data. The mitochondrial genome assembly graph was visualized using Bandage, followed by the manual removal of chloroplast and nuclear genome-derived single contigs [24]. Subsequently, the Nanopore data were aligned with the graphical mitochondrial genome fragments using BWA (0.7.17-r1188) software, with the resultant Nanopore data then being applied to resolve repetitive regions within the graphical plant mitochondrial genome [25]. Finally, we obtained the multi-branch mitochondrial genome of *C. asiatica*.

The mitochondrial genome’s PCGs were annotated using *Arabidopsis thaliana* as the reference genome with the GeSeq [26]. Transfer RNAs (tRNAs) were annotated using tRNAscan-SE, and ribosomal RNAs (rRNAs) were identified through BLASTN [27,28]. All annotation errors in the mitochondrial genome underwent manual correction using Apollo [29]. Finally, the annotated sequences were deposited in GenBank under accession numbers PV739434-PV739436.

### 2.3. Codon Usage Analysis and Repetitive Sequence Identification

Protein-coding sequences were isolated from the genome using Phylosuite (version 1.1.16) [30]. Codon usage bias analysis of mitochondrial PCGs was conducted using MEGA (version 7.0), along with the calculation of relative synonymous codon usage (RSCU) values [31]. The simple sequence repeats (SSRs), tandem repeats (TRs), and dispersed repeats were detected using MISA (version 2.1) (https://webblast.ipk-gatersleben.de/misa/, accessed on 2 July 2022), TRF (version 4.09) (https://tandem.bu.edu/trf/trf.unix.help.html, accessed on 2 July 2022), and the REPuter web server (https://bibiserv.cebitec.uni-bielefeld.de/reputer/, accessed on 2 July 2022) [32,33,34]. The results were visualized using Circos (version 0.69-9) [35].

### 2.4. Mitochondrial-to-Plastid Sequence (MTPTs) Transfer Analysis

Homologous sequences between the mitochondrial and chloroplast genomes were identified through BLASTn (v2.13.0) [28]. The results were visualized using the Circos package (v0.69-9) [36].

### 2.5. Phylogenetic and Synteny Analysis

We retrieved the mitochondrial genomes of species from NCBI (https://www.ncbi.nlm.nih.gov; accessed on 1 August 2022). Shared genes were retrieved using PhyloSuite (v1.1.16) [30]. MAFFT (v7.505)-based sequence alignment preceded MrBayes-mediated Bayesian phylogeny construction, to be used in maximum likelihood phylogenetic reconstruction (IQ-TREE; Minh et al. [34]) under the GTR+F+R2 model. Tree robustness was assessed with 1000 ultrafast bootstrap replicates, and final visualization was performed in iTOL [37,38].

To investigate the synteny of mitochondrial genomes, conserved homologous sequences between *C. asiatica* and its related species were identified using BLASTn with the following parameters: -evalue 1e-5, -word_size 9, -gapopen 5, -gapextend 2, -reward 2, and -penalty 3 [28]. Only syntenic blocks with minimum lengths of 500 bp were selected for further analysis. Multiple synteny plots were generated using MCscanX [39].

### 2.6. Prediction and Identification of RNA Editing Sites

We collected the roots, stems, and leaves of *C. asiatica* as materials for lncRNA detection, with three biological replicates each. LncRNA sequencing was performed by Benagen (Wuhan, China), with quality control filtering applied. We then aligned the transcriptome sequencing data to protein-coding genes (PCGs) using TopHat2 [40], permitting a maximum of 7 nucleotide mismatches. Subsequently, potential RNA editing events were identified by comparing the mitochondrial DNA and RNA alignments using REDItools [41]. Editing sites were filtered at a minimum coverage depth of 100× and an editing frequency threshold not lower than 0.10 [42]. RNA editing sites in 33 unique PCGs from the *C. asiatica* mitochondrial genome were predicted using the PREPACT (http://www.prepact.de/), with a cutoff value set at 0.2 [43]. Tissue-specific RNA editing sites in the roots, stems, and leaves were further identified using lncRNA sequencing data.

## 3. Results

### 3.1. General Features of the C. asiatica Mitochondrial Genome

**Figure 1:**
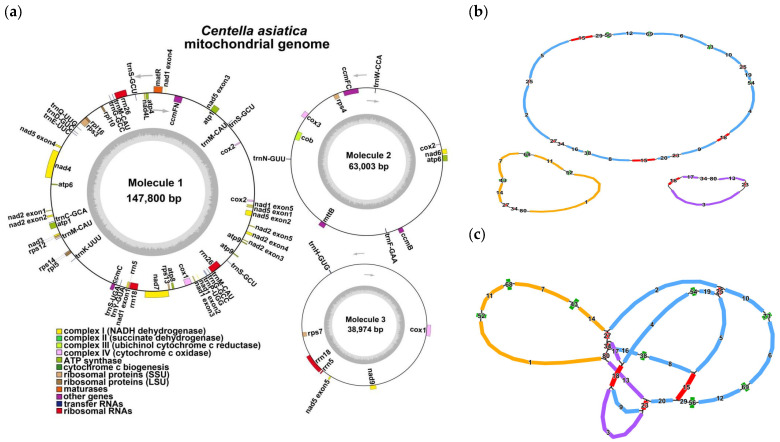
The structure of the *C. asiatica* mitochondrial genome. (**a**) Circular maps of the *C. asiatica* mitogenome; (**b**) master circular structure; (**c**) mitogenome assembly graph and possible connections. In panels (**b**,**c**), red nodes flag any computationally predicted repetitive elements, whereas green nodes designate putative chloroplast-to-mitochondrion-transferred sequences. Molecular markers 1–3 are denoted by blue, orange, and purple nodes, respectively.

We assembled a multibranched mitochondrial genome of *C. asiatica* (Figure 1 and Table S1 in the Supplementary Materials). After resolving the issue of repetitive regions using Nanopore data, we obtained three primary circular contigs with a total length of 249,777 bp and a GC content of 45.46% (Figure 1a and Table 1). We observed the following: molecule 1 contains five repetitive regions (contig15, 18, 25, 27, and 34); molecule 2 contains fragments 27 and 34; and molecule 3 contains fragments 18, 23 and 34. We observed that molecule 1 contains contig 15, contig 18, contig 25, contig 27, and contig 34; molecule 2 contains contig 27 and contig 34; while molecule 3 contains contig 18, contig 23, and contig 34. Notably, molecule 1 carries two non-adjacent copies of contig 15 and contig 18, respectively (Figure 1b,c). Based on the assembly structure, we annotated a total of 51 genes, including 33 PCGs (including 24 kinds of unique mitochondrial core genes and 9 non-core genes), along with 15 tRNA genes and 3 rRNA genes. The core genes included: 5 ATP synthase genes (*atp1*, *atp4*, *atp6*, *atp8*, and *atp9*); 9 NADH dehydrogenase genes (*nad1*, *nad2*, *nad3*, *nad4*, *nad4L*, *nad5*, *nad6*, *nad7*, and *nad9*); 4 cytochrome c biogenesis genes (*ccmB*, *ccmC*, *ccmFc*, and *ccmFn*); 3 cytochrome c oxidase genes (*cox1*, *cox2*, and *cox3*); 1 membrane transport protein gene (*mttB*); 1 maturase gene (*matR*); and 1 ubiquinol-cytochrome c reductase gene (*cob*). The non-core genes included three large ribosomal subunit genes (*rpl5*, *rpl10*, and *rpl16*) and six small ribosomal subunit genes (*rps3*, *rps4*, *rps7*, *rps12*, *rps13*, and *rps14*). Some genes exist as multiple copies, while others are a single copy (see Table S2 for details).

### 3.2. Codon Usage Bias in PCGs

Codon usage bias was performed on the 33 unique PCGs identified within the *C. asiatica* mitochondrial genome (Figure S1 and Table S3). Histidine (His) showed the strongest codon usage bias with CAU (RSCU = 1.52), followed by glutamine (Gln) preferring CAA (RSCU = 1.51). Notably, phenylalanine (Phe) exhibited the lowest bias, with maximum RSCU values of <1.20.

### 3.3. Repetitive Sequences

A total of 109 dispersed repeats, 26 TRs, and 48 SSRs were identified in *C. asiatica* (Tables S4–S6).

**Figure 2:**
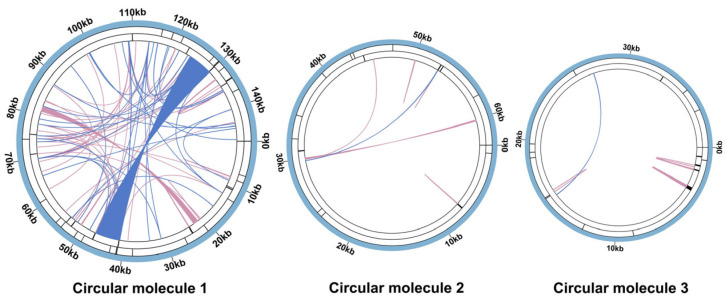
Mitochondrial repeat sequences of *C. asiatica*. The colored lines on the innermost circle connect the two repeat units of the dispersed repeats, with blue lines representing palindromic repeats and pink lines indicating forward repeats. TRs are indicated by black segments on the secondary concentric ring, while the outermost ring displays black segments corresponding to SSRs.

In circular molecule 1, repetitive sequences were the most abundant. We identified 90 pairs of dispersed repeats, comprising 53 forward repeats and 37 palindromic repeats. We identified zero instances of reverse/complementary repeats. Intriguingly, we identified an exceptionally long palindromic repeat spanning 5870 bp, a feature rarely observed in characterized plant mitochondrial genomes (Figure 2 and Table S4). Additionally, 13 TRs were annotated (Table S5), and 27 SSRs were detected. Monomeric and dimeric SSRs collectively constituted nearly half of them (44.44%). Specifically, two monomeric SSRs were identified: an adenine (*A*) monomer repeat and a thymine (*T*) monomer repeat (Table S6). Among the dimeric SSRs, *TA* repeats represented the most prevalent type, accounting for approximately 50.00% of all dimeric SSRs.

In circular molecule 2, we identified nine pairs of dispersed repeats, comprising seven forward repeats and two palindromic repeats. Similarly, we identified zero instances of reverse/complementary repeats in this molecule. The maximum lengths observed were 52 bp for forward repeats and 31 bp for palindromic repeats (Table S4). Five tandem repeat regions were annotated (Table S5), and ten simple sequence repeats (SSRs) were detected. Forty percent of the identified SSRs originated from monomeric and dimeric configurations. Only one monomeric SSR type was identified—thymine (*T*). *TA* repeats also predominated in dimeric SSRs, accounting for 66.67% of the total (Table S6).

A total of 10 dispersed repeat pairs were identified in circular molecule 3, consisting of 9 forward and 1 palindromic repeat. Reverse and complementary repeats remained undetected throughout the analysis. The longest forward repeat measured 42 bp (Table S4). Eight tandem repeat regions were annotated (Table S5), and eleven simple sequence repeats (SSRs) were detected, including one dimeric SSR (an *AT* repeat). Trimeric and tetrameric SSRs collectively accounted for 72.73% of all SSRs. Notably, neither monomeric nor hexameric SSRs were observed in circular molecule 3 (Table S6).

### 3.4. Sequence Transfer and Synteny Analysis

**Figure 3:**
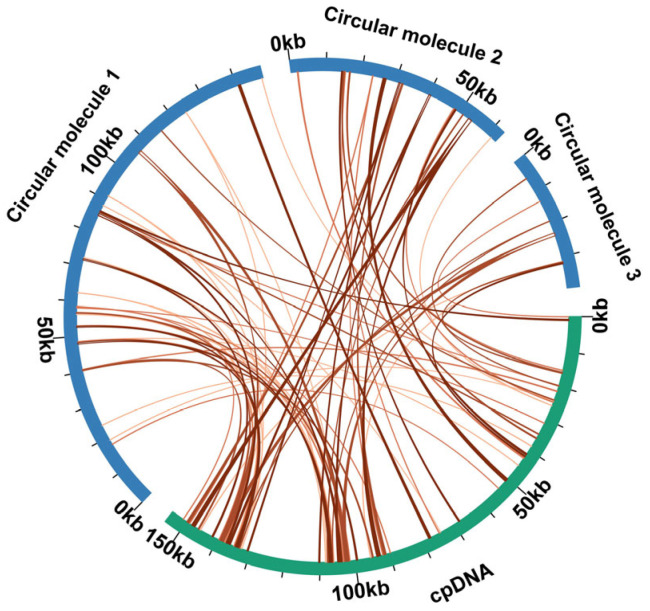
MTPT transfer events in *C. asiatica*. The blue curvilinear segments denote the mitogenome, the green arcs denote the chloroplast genome, and the orange connecting lines between the arcs indicate homologous genomic sequences.

Comparative analysis between the chloroplast and the mitochondrial genomes was conducted to identify MTPTs. Sequence homology analysis (Table S7) revealed 86 homologous sequences between the *C. asiatica* mitochondrial and chloroplast genomes, with those sequences totaling 21,166 bp in length and comprising 8.47% of the complete mitochondrial genome. Among these, two fragments exceeded 1000 bp in length, with both located on circular molecule 2 (1088 bp each). MTPTs exhibit a relatively uniform distribution across the mitochondrial genome but display significantly higher density in the two inverted repeat (IR) regions of the chloroplast genome (Figure 3). This distribution pattern is consistent with recent observations in the mitochondrial genome of *Uncaria* [44]. Annotation of these homologous sequences identified 5 complete genes, including 1 protein-coding gene (*petG*) and 4 tRNA genes (*trnD-GUC*, *trnH-GUG*, *trnN-GUU*, and *trnW-CCA*).

**Figure 4:**
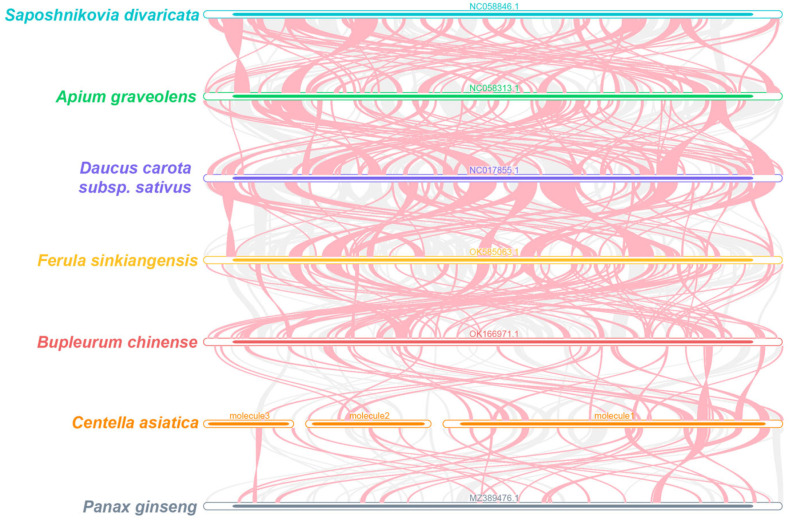
*C. asiatica* mitochondrial genome synteny. These bars signify the mitochondrial genomes, with the homologous sequences connected by lines. The red areas indicate inversions, while the gray areas have high homology. Blocks of less than 0.5 kb being shared between species are not shown.

The multiple synteny plots between *C. asiatica* and other Apiaceae species were generated using MCscanX (Figure 4). The numbers of the collinear blocks were detected, although with short average lengths. We also identified species-specific non-homologous regions in *C. asiatica*. These results reveal the divergent arrangements of collinear blocks among Apiaceae mitochondrial genomes, which are indicative of frequent genomic rearrangements between *C. asiatica* and its relatives.

### 3.5. Phylogenetic Evolution

**Figure 5:**
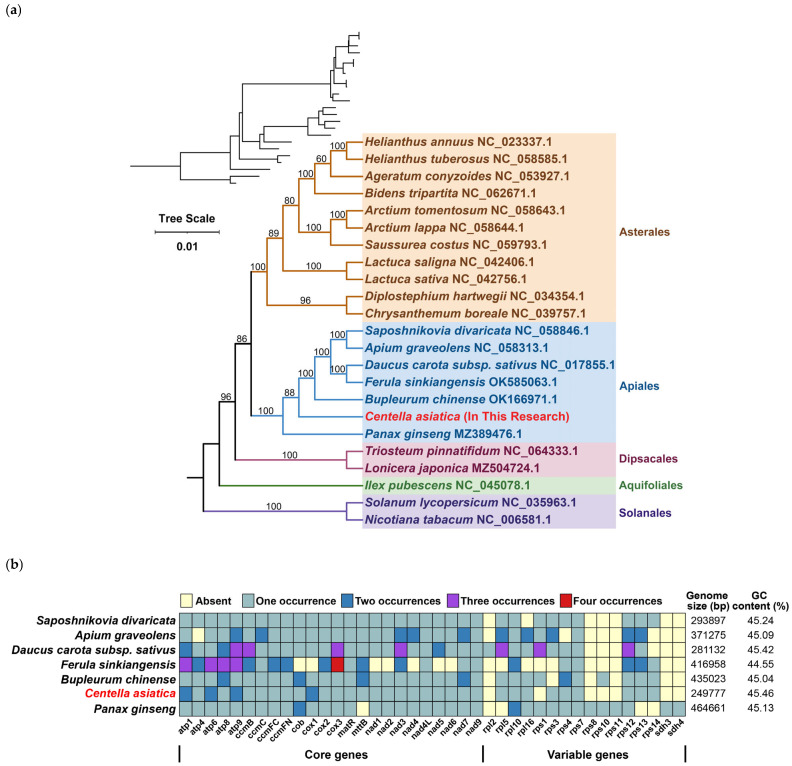
Phylogenetic and gene characteristics of the mitochondrial genome of *C. asiatica*. (**a**) Phylogenetic tree. (**b**) Variations in genes, genome sizes, and GC values in mitochondrial genomes of seven Apiales species.

Phylogenetic analysis employed 20 evolutionarily stable mitochondrial PCGs (*atp1*, *atp4*, *atp6*, *atp8*, *ccmC*, *ccmFc*, *ccmFn*, *cox1*, *cox2*, *cox3*, *matR*, *nad1*, *nad2*, *nad3*, *nad4L*, *nad5*, *nad7*, *nad9*, *rps12*, and *sdh4*) across 23 species from five angiosperm orders. We designated two Solanales as outgroups. The tree strongly supported a recent phylogenetic relationship between *C. asiatica* and *Bupleurum chinense*, and *C. asiatica* was located in an early branch of Apiales (Figure 5a).

Concurrently, we analyzed the copy number distributions of these 20 genes in seven species, including *C. asiatica* (Figure 5b), revealing significant interspecies variation. Among the core genes, *Saposhnikovia divaricata*, *Daucus carota* subsp. *sativus*, *B. chinense*, and *C. asiatica* maintained more complete sets of core genes, although with varying copy numbers: *D. carota* subsp. *sativus* possessed the highest overall copy number, followed by *C. asiatica* and *B. chinense*, while *S. divaricata* consistently maintained single-copy status for all genes. In contrast, non-core genes (e.g., *rpl* and *rps*) exhibited irregular copy number patterns without discernible regularity.

### 3.6. RNA Editing Analysis in C. asiatica

#### 3.6.1. RNA Editing Prediction

**Figure 6:**
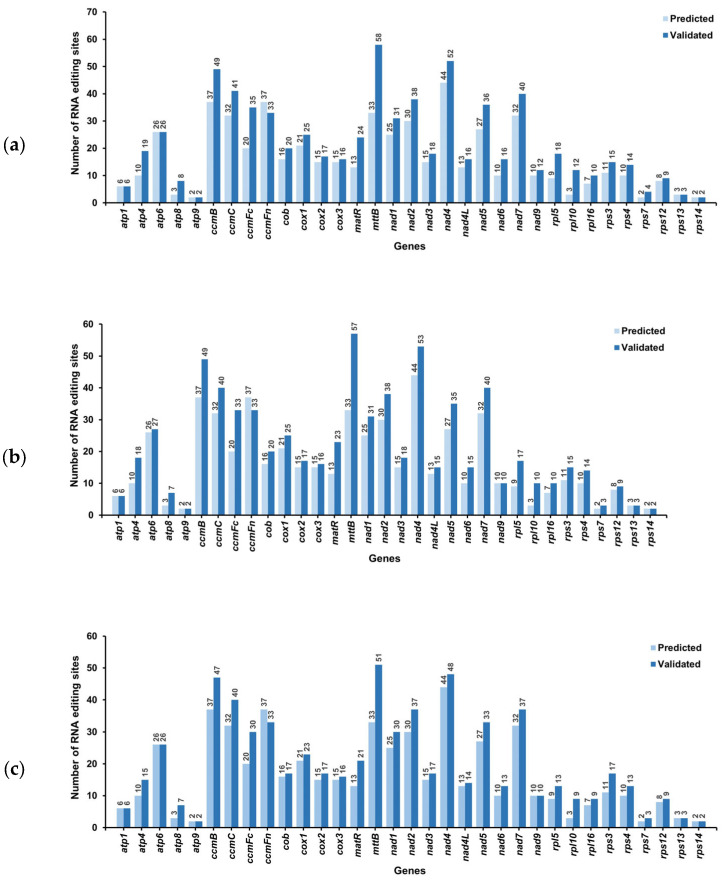
Prediction and tissue-specific identification of RNA editing sites in *C. asiatica*. (**a**) RNA editing sites in the roots; (**b**) RNA editing sites in the stems; (**c**) RNA editing sites in the leaves.

We predicted a total of 547 RNA editing sites in the 33 mitochondrial PCGs through computational methods (Figure 6), all involving *C*-to-*U* conversions. RNA editing analysis identified 44 modification sites in the nad4 gene, thereby representing the highest density among all PCGs, followed by *ccmB* and *ccmFn* (37 sites each). The fewest editing sites were observed in *atp9*, *rps7*, and *rps14* (two sites each).

#### 3.6.2. High-Throughput Verification of RNA Editing Sites

To systematically identify these potential RNA editing sites, we examined mitochondrial LncRNA-seq data from root, stem, and leaf samples for all 33 PCGs. A total of 725, 711, and 668 editing sites were detected in the tissues of root, stem, and leaf samples, respectively (Figure 6).

**Figure 7:**
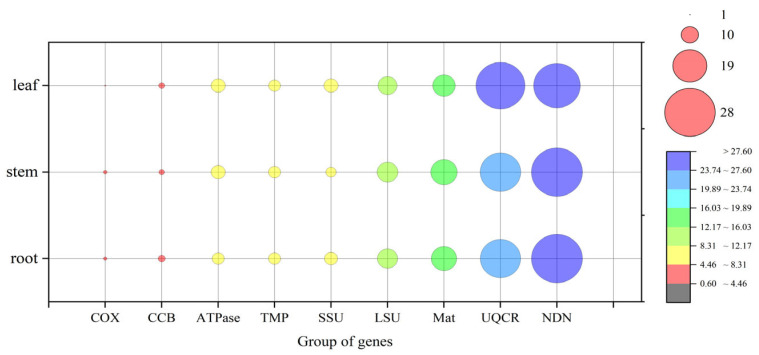
Distribution of unpredicted RNA editing across different gene groups.

Comparison between the experimentally observed RNA editing sites and predicted sites revealed 514, 515, and 513 confirmed editing sites in the samples from roots, stems, and leaves, respectively (Figure 6). Notably, we additionally identified 211 unpredicted editing sites in roots, 196 in stems, and 155 in leaves. These unpredicted editing sites predominantly occurred in NADH dehydrogenase genes, maturases, ubiquinol-cytochrome c reductase genes, and small ribosomal subunit genes (Figure 7 and Table S8).

As shown in Table S10, mitochondrial RNA editing induced distinct codon substitution patterns across *C. asiatica* roots, stems, and leaves. We identified 9 types of synonymous substitutions involving Ile (isoleucine), Phe (phenylalanine), Val (valine), Thr (threonine), Ser (serine), Leu (leucine), Tyr (tyrosine), Ala (alanine), and Asn (asparagine). Additionally, Asp synonymous substitutions were detected in the roots and stems, while Ala synonymous substitutions were found in the leaves.

What is more striking is the base substitutions that alter the codons. In roots, 628 of 725 RNA editing sites (86.6%) resulted in non-synonymous substitutions, encompassing 11 amino acid conversions. Pro-to-Leu and Ser-to-Leu substitutions predominated, collectively accounting for approximately 47% of all non-synonymous substitutions in the roots and stems, with a slightly lower proportion in the leaves (36%; Table S9).

Codon editing-induced premature termination codons (PTCs) and de novo promoter formation could disrupt key physiological processes in plants. Root tissues harbored PTCs at seven mitochondrial loci: *atp6-240*, *atp9-75*, *ccmFC-429*, *nad1-73*, *rpl15-152*, and *rpl6-13/63*. In stems, PTCs were detected at *atp6-240*, *atp9-223*, *ccmFC-429*, *nad1-73*, *rpl16-13/63*, and *rpl5-152*, whereas leaf tissues contained PTCs at *atp6-240*, *atp9-75*, *ccmFC-429*, and *rpl16-13/63*. Notably, PTCs in *rpl15* and *rpl16* (spanning both 5′ and internal regions) showed tissue-independent conservation. Conversely, tissue-specific initiation sites emerged at *cob-387*, *cox2-148*, *nad7-75*, and *rpl16-135* in the roots; sites at *cob-384*, *cox2-148*, *nad7-75*, and *rpl16-135* emerged in the stems; and sites at *cob-387*, *cox2-148*, and *nad7-75* emerged in the leaves.

## 4. Discussion

### 4.1. The Functional Association Between Genome Topology and Repetitive Sequences

The assembly of plant mitochondrial genomes presents challenges, due to their structural diversity and high repetitive sequence content. Plant mitochondrial genomes exhibit various configurations, including linear, circular, branched, and multimeric circular structures [45,46,47]. The *C. asiatica* genome displays a notably branched structure (Figure 1c), with a total length of 249,777 bp and a GC content of 45.46%. Assembly revealed that it consists of three circular contigs (Figure 1a,b). This complex topology is closely associated with the high abundance of repetitive sequences within the genome [9,48]. We identified 109 dispersed repeats, 26 TRs, and 48 SSRs in the *C. asiatica* mitochondrion. These repeats, especially those fragments shared among different circular molecules (e.g., contigs 18, 27, and 34), are key drivers of intramolecular homologous recombination, leading to the formation of a multipartite structure.

Particularly noteworthy is our discovery of a large palindromic repeat spanning 5870 bp in molecule 1 (Figure 2). Such a long repeat is rare among the reported plant mitochondrial genomes and provides a strong molecular basis for genomic instability, likely mediating frequent recombination events such as inversions, deletions, or duplications [46,48]. This is consistent with the poor synteny that we observed among Apiaceae species (Figure 4), indicating that the *C. asiatica* mitochondrial genome has undergone drastic rearrangements and exhibits a high degree of evolutionary dynamism. Furthermore, the differences in SSR types among the circular molecules (e.g., mono- and di-nucleotide repeats dominating in molecules 1 and 2, versus the tri- and tetra-nucleotide repeats dominating in molecule 3) also suggest that different genomic regions may be subject to distinct microevolutionary pressures.

### 4.2. MTPTs and Mitochondrial Functional Autonomy

Gene transfer between the nucleus and organelles constitutes the molecular basis of co-evolution through nuclear–organelle interactions. This study found that the proportion of MTPTs in the *C. asiatica* mitochondrial genome reaches 8.47% (21,166 bp), a ratio that is significantly higher than that in most reported Apiaceae species, such as *B. chinense* and *Angelica biserrata* (typically 2–4%) [10,49,50]. These transferred fragments include not only a complete protein-coding gene (*petG*) but also four functional tRNA genes.

The transfer of tRNA genes is particularly significant, as the native set of tRNAs encoded by plant mitochondria is often insufficient to support the translation of all codons. “Borrowing” tRNA genes from the chloroplast can compensate for deficiencies in the mitochondrial translational machinery and optimize protein synthesis efficiency, a mechanism discussed elsewhere in the context of other medicinal plants [51]. The widespread distribution of these MTPTs in the *C. asiatica* mitochondrial genome, with their origins concentrated in the IR regions of the chloroplast genome—a known hotspot for DNA insertions [52]—further confirms the activity of this inter-organellar communication. The presence of a high proportion of MTPTs likely endows the *C. asiatica* mitochondrion with greater genetic and functional autonomy to meet its specific physiological and metabolic demands. However, this hypothesis still requires further functional genomic and biochemical validation.

### 4.3. Tissue-Specific Regulation of RNA Editing

RNA editing is a critical post-transcriptional modification in plant mitochondria that alters the sequence of RNA transcripts, often by converting cytidines to uridines [53]. This study systematically validated RNA editing events in three tissues of *C. asiatica*—root, stem, and leaf tissues—and found significant tissue specificity. The total number of editing sites was highest in the root (725) and lowest in the leaf (668), indicating that different tissues have varying requirements for the “repair” or “recoding” elements of mitochondrial gene transcripts [14,15].

#### 4.3.1. Tissue-Biased Amino Acid Substitutions

Non-synonymous substitutions from proline (Pro) to leucine (Leu) and from serine (Ser) to leucine (Leu) were predominant in roots and stems (accounting for ~47% of substitutions) but were less frequent in leaves (36%). Leucine is a hydrophobic amino acid, and this high-frequency substitution may alter the hydrophobic core or transmembrane domains of mitochondrial proteins, particularly those of the respiratory chain complexes, thereby modulating their activity or stability in different tissues [54]. As supporting and absorptive organs, roots and stems have different energy demands and stress responses compared to leaves, which are primarily photosynthetic. This editing divergence is likely an adaptation to functional tissue differentiation.

Although this study used roots, stems, and leaves as experimental units and revealed significant inter-organ differences in RNA editing—which may represent a macroscopic manifestation of ‘tissue specificity’—we acknowledge that each organ comprises over 10 distinct cell types with functionally and ultra-structurally heterogeneous mitochondria. Due to current technical limitations, we could not dissect the relative contributions of individual cell types to the observed editing signals. Future studies employing single-cell sequencing or laser microdissection could further examine cell type-specific RNA editing patterns, thereby refining our understanding of the post-transcriptional regulatory network in *C. asiatica* mitochondria.

#### 4.3.2. Tissue-Specific Formation of PTC

We identified the tissue-specific PTCs in our samples. For instance, the root-specific editing seen at the *nad1-73* site is predicted to generate a truncated polypeptide at the *N*-terminus, which would likely disrupt the assembly of mitochondrial respiratory complex I and downregulate its function. This mechanism, where RNA editing creates a functional stop codon from a sense codon, has been shown to be critical for producing functional proteins or, conversely, for generating truncated products that are associated with specific phenotypes, such as cytoplasmic male sterility in maize [55].

#### 4.3.3. Tissue-Specific Formation of Start Codons

Editing events also created new translation initiation sites in a tissue-specific manner (e.g., at *rpl16-135*), which could produce protein isoforms with different N-terminal sequences, while allowing for fine-tuned functional regulation.

### 4.4. Numerous Unpredicted Editing Sites Reveal the Uniqueness of the RNA Editing Machinery in C. asiatica

A key finding of this study is that a substantial number of RNA editing sites (23–29%) in all tissues could not be predicted using existing bioinformatics tools [43]. These unpredicted sites were predominantly located in genes for NADH dehydrogenase, *matR*, and ribosomal proteins (Figure 7). This result perhaps suggests that current prediction algorithms, which are based on conservation models derived primarily from model plants like *Arabidopsis*, fail to capture all the recognition signals for editing sites in *C. asiatica*. Studies in tobacco mitochondria have found that it may possess species-specific or tissue-specific RNA editing regulatory mechanisms, such as unique PPR proteins that recognize atypical cis-acting elements [20,56]. This may provide an insightful clue, but experimental evidence in the literature on our study species is currently lacking. Whether the unexpectedly discovered editing events share similar mechanisms with those found in tobacco requires further functional validation. However, these unpredicted sites may represent a valuable resource for discovering novel editing factors and recognition motifs.

In summary, this study reveals that through complex structural rearrangements, frequent inter-organellar gene transfer, and sophisticated tissue-specific RNA editing, the *C. asiatica* mitochondrial genome has formed a unique genetic and regulatory network.

## 5. Conclusions

Using Illumina short-read and Nanopore long-read sequencing data, we completed the assembly and annotation of the *C. asiatica* mitochondrial genome. Our comprehensive analysis included structural characterization, repetitive sequence annotation, gene content profiling, RNA editing site prediction and identification, homologous sequence analysis, collinearity assessment, and phylogenetic reconstruction. By comparing computational predictions with experimental RNA editing data, we revealed tissue-specific editing patterns in the tissue samples from roots, stems, and leaves, and found that in different tissues, some core genes (such as ATP synthase, panthenol cytochrome c reductase, ribosomal large subunit, cytochrome c biogenesis, and cytochrome c oxidase) were truncated or retranslated; the incidence of these events in leaf tissues was lower than that in other tissues. These findings will serve as a valuable resource for future studies on the genetic evolution and mitochondrial gene expression of this species.

## Figures and Tables

**Table 1:** Summary of the major characteristics of the *C. asiatica* mitochondrial genome.

NCBI Accession Number	Contigs	Type	Length	GC Content
PV739434-PV739436	Molecule 1–3	Branched	249,777 bp	45.46%
PV739434	Molecule 1	circular	147,800 bp	46.15%
PV739434	Molecule 2	circular	63,003 bp	44.38%
PV739434	Molecule 3	circular	38,974 bp	44.65%

## Data Availability

The datasets presented in this study can be found in online repositories. The names of the repository/repositories and accession number(s) can be found in the article/Supplementary Materials.

## Supplementary Materials

The following supporting information can be downloaded at: https://www.mdpi.com/article/10.3390/genes16080953/s1, Figure S1: Codon usage bias in *Centella asiatica*; Table S1: Path selection solutions for repetitive regions resolved by Oxford analysis; Table S2: Mitochondrially encoded genes in *Centella asiatica*; Table S3: Codon usage bias in *Centella asiatica* mitochondria; Table S4: Dispersed repeats sequences in the mitochondrial genome of *Centella asiatica*; Table S5: Tandem repeat sequences in the mitochondrial genome of *Centella asiatica*; Table S6: SSRs in the mitochondrial genome of *Centella asiatica*; Table S7: The homologous DNA fragment in the *Centella asiatica* mitochondrial genome; Table S8: The homologous DNA fragment in the *Centella asiatica* mitochondrial genome; Table S9: Codon substitution profile in mitochondrial DNA editing of *Centella asiatica* root, stem, and leaf samples; Table S10: Tissue specificity landscapes of RNA editing-derived promoters and terminators in *Centella asiatica* roots, stems, and leaves.
