# Supplementary figures and images for: Mitochondrial Genome and RNA Editing Tissue Specificity of Centella asiatica
Source: Genes (Basel). 2025 Aug 12;16(8):953. doi: 10.3390/genes16080953 (PMC12385686; doi:10.3390/genes16080953)

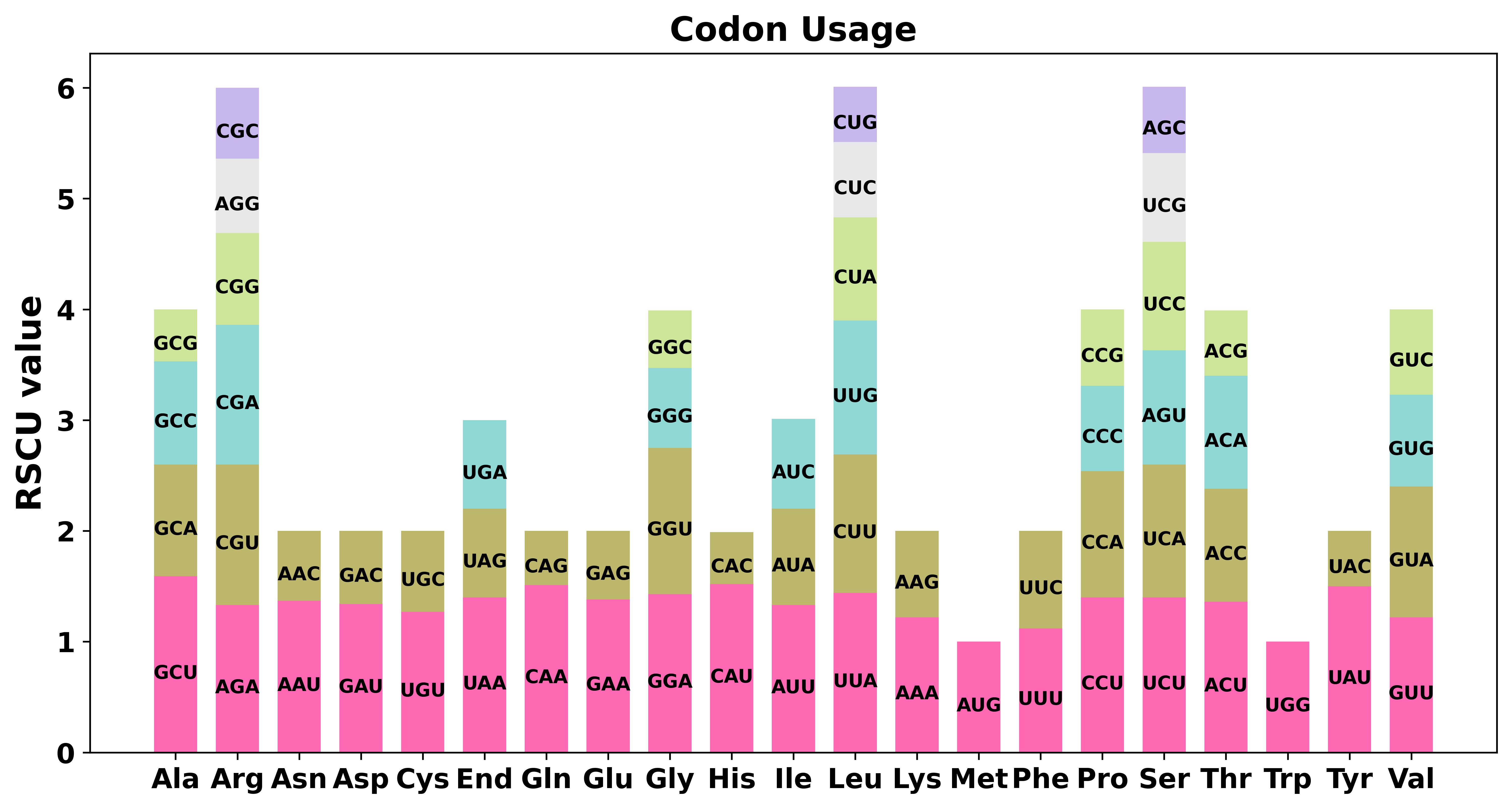

Supplement: Supplementary file 1 [file genes-16-00953-s001.zip › Supplementary Material/Supplementary Material 1 - Figure S1/Figure S1Codon usage bias in Centella asiatica.jpg]
